# Non-urgent accident and emergency department use as a socially shared custom: a qualitative study

**DOI:** 10.1136/emermed-2014-204039

**Published:** 2015-04-04

**Authors:** Simone Keizer Beache, Cornelia Guell

**Affiliations:** 1 Public Health Group, Faculty of Medical Sciences, University of the West Indies, Bridgetown, Barbados; 2 MRC Epidemiology Unit, University of Cambridge, Cambridge, UK

**Keywords:** emergency department, primary care

## Abstract

**Objective:**

We explored attitudes of non-urgent accident and emergency department (AED) patients in the middle-income healthcare setting Saint Vincent and the Grenadines (SVG) in the Caribbean to understand how and why they decide to seek emergency care and resist using primary care facilities.

**Methods:**

In 2013, we conducted 12 semistructured interviews with a purposive sample of non-urgent AED users from a variety of social backgrounds. Verbatim transcripts were analysed with a grounded theory approach.

**Results:**

In this study, we found, first, that participants automatically chose to visit the AED and described this as a locally shared custom. Second, the healthcare system in SVG reinforced this habitual use of the AED, for example, by health professionals routinely referring non-urgent cases to the AED. Third, there was also some deliberate use; patients took convenience and the systemic encouragement into account to determine that the AED was the most appropriate choice for healthcare.

**Conclusions:**

We conclude that the attitudes and habits of the Vincentian non-urgent patient are major determinants of their AED use and are intricately linked to local, socially shared practices of AED use. Findings show that health services research should reconsider rational choice behaviour models and further explore customs of health-seeking.

**Key messages**

**What is already known on this subject?**

- Crowding of accident and emergency departments is a worldwide phenomenon, often attributed to non-urgent use of the facilities. Research to date found that reasons include inadequate access to and dissatisfaction with primary care facilities, as well as patients’ erroneous perceptions of urgent complaints. Increasing patient education and facilities does not appear to reduce non-urgent use in many settings.

**What might this study add?**

- This qualitative study conducted among emergency department users in the Caribbean suggests that non-urgent accident and emergency use can be habitual and socially shared and encouraged. Health service researchers should re-examine widely used rational choice constructs and instead explore habit formation and the social context of healthcare-seeking.

Throughout the world, accident and emergency departments (AEDs) are noted to be overcrowded and compromised in their principal care role of providing urgent life and function saving health services.^1–4^ Crowding in AEDs has many consequences beyond increasing patient waiting times, such as general patient dissatisfaction, lowered healthcare provider productivity, and most importantly, potentially prolonging the time to care for critically ill patients, resulting in poor outcomes.^3^ ^5^

One proposed reason for crowding is the inappropriate use of these departments by persons who do not have a medically urgent complaint.^6^ Key in the decision-making process of prospective non-urgent patients seems to be their attitudes and perceptions relative to their ailment and the health system.^7–9^ Studies found that convenience and accessibility of emergency facilities, diagnostics and prescription drugs, and cost and dissatisfaction with caregivers in primary care facilities were the major reasons for non-urgent AED visits.^8^ ^10–12^ Researchers also found that the patients’ understanding of the role of the emergency healthcare services and their perceptions of urgent and non-urgent complaints differ significantly from those of healthcare providers,^5^ ^13^ ^14^ reporting great anxiety and need for urgent medical attention.^15^ ^16^ The findings of these studies suggest that a deeper understanding of these perceptions and attitudes should facilitate the development of tailored interventions that are more likely to be effective at reducing the non-urgent use of the AED.

AED crowding becomes critical when emergency care is provided in a resource-poor developing country setting where little research has yet been conducted on this topic^17^ and in small healthcare systems. Saint Vincent and the Grenadines (SVG), a multi-island, middle-income Eastern Caribbean nation of about 103 000 inhabitants,^18^ has only one full service hospital, a 205 bed facility with one 11-bed AED. In 2012, the AED had 25 395 patient visits; 17.5% of these visits required admission and only 0.65% were assessed as critical.^19^ Many of the AED patients are repeat visitors with non-urgent complaints who bypass the primary care district clinic located next door to the AED. SVG, as other larger and richer countries, attempted to address the inappropriate use of the AED by the establishment of a fast-track service as an alternative at this primary care facility with little success, as was reported elsewhere.^20^ The failure of these interventions is proposed to be partially based on not considering some key factors that might determine the use of the AED.

Therefore, in this research we employed a qualitative research approach to explore the attitudes that influence the decision-making of the Vincentian non-urgent AED patients. Our particular focus was to investigate healthcare-seeking behaviour as it plays out in a middle-income setting and to interrogate if common health service use models are useful.^21^

## Methods

The only emergency facility of SVG was chosen as the setting of this study. Public healthcare is provided free at the point of access to all persons younger than 17 years and older than 60 years, and is heavily subsidised to those 17–60 years old. There are about 40 primary care clinics spread across the 150 square miles of the multi-island state of SVG aimed at providing easy access to healthcare to all inhabitants, and one secondary care hospital with its AED in the capital city of Saint Vincent.

We selected a purposive sample of adult female and male participants of different ages from a variety of social backgrounds to represent a diverse set of perspectives and experiences. The researcher (SK-B) obtained permission to select patients by looking at their admission cards that gave their demographics and triage category of complaint. The researcher approached only patients who had been triaged as non-urgent by the AED nurse on the days of data collection and expressed an interest to participate in the study. Purposive sampling meant that patients were not approached if particular sample criteria have already been saturated, for example, men, middle-aged, manual occupation. Twelve patients were interviewed in a quiet room in the AED from May to June 2013 on the same day they were approached. The sample (see table 1) consisted of seven men and five women between the ages of 19 and 72 years. All but two participants had non-trauma-related complaints. Five participants had complaints that began in the preceding 24 h. None of the participants had been referred to the AED on this occasion. Seven persons reported having visited the department previously on multiple occasions. Participants came from communities on the leeward and windward areas of the island Saint Vincent as well as Kingstown, and included labourers, homemakers, civil servants and retired persons.

**Table 1:** Profile of participants

No.	Sex	Age	Kingstown resident	Occupation	Day and time of presentation	Patterns of AED use	Presenting complaint	Final diagnosis
1	M	72	Yes	Driver	Wednesday am	Frequent	Lower back pain	Sciatica
2	F	33	No	Housekeeper	Thursday pm	Frequent	Vaginal bleed	Metorrhagia
3	M	24	No	Farmer	Friday am	First visit	Bump on cheek	Sebaceous cyst
4	M	53	No	Mechanic	Friday am	Previous visit	Shoulder pain	Bursitis
5	F	48	No	Housewife	Friday am	Rare use	Headache	Migraine
6	F	19	No	Student	Monday am	Previous visit	Abdominal pain	Dysmenorrhoea
7	M	25	No	Security officer	Monday am	Previous visit	Pain with urination	Urethritis
8	M	63	No	Unemployed security officer	Tuesday pm	Rare use	Rash	Eczema
9	M	37	No	Labourer	Tuesday pm	First use	‘Chest burn’	Dyspepsia
10	M	21	No	Labourer	Wednesday am	Previous visit	Scorpion bite	Uncomplicated scorpion bite
11	F	50	No	Civil servant	Thursday pm	Previous visit	Dizziness	Benign labrynthitis
12	F	46	No	Clergy	Thursday pm	Previous visit	Swollen foot	Sprained ankle

AED, accident and emergency department.

Semistructured interviews were conducted using an interview guide appropriate for this setting, designed drawing from the Andersen Model of assessing health services use^21^ and from a guide created by Durand *et al*^8^ Interviews lasted up to 35 min and asked open questions about their current health complaint, reasons for seeking help at the AED, usual healthcare-seeking practices and views on the AED and other available healthcare facilities (see box 1).

Box 1 **Interview guide**

1. Can you describe to me what happened to make you come to AED today?
2. Why did you choose to come to the emergency department instead of the district clinic or your private doctor?
  - Why do you feel/believe/think this way?
  - What has been your experience with the casualty?
  - Did anyone else tell you to come here?
  - What do people feel/think/believe about the casualty?
3. What do you usually do when you get sick?
  - Do you go to the district clinic?
  - What do you go to the district clinic for?
  - Do you go to a private doctor? What for?
4. Do you think the emergency department is the best place to treat your current health problems?
  - What is the function of the emergency department?
  - What is the function of the district clinic?
  - What do you understand when I say ‘an emergency' or 'urgency'?
  - What do you think is good service?
5. What do you think about the emergency department as compared with other healthcare facilities?
  - Why do you think that?
  - What has been your experience with other health facilities?
  - What do you think about the staff at the casualty and the district clinics and the private clinics?
  - What do you think about their training, qualifications and characteristics?
  - What do you think about the health facilities available?
6. What do you think about paying for healthcare?
  - Would you be willing to pay for service at the AED or the district clinics?
  - How do you feel about the cost of healthcare in Saint Vincent?
7. What would you do if you got sick in Georgetown?
  - Would you travel to Kingstown to be treated or would you seek care in Georgetown?
  - What would make you come to town or stay in Georgetown?
8. What do you think about the healthcare that is available in Saint Vincent and the Grenadines (SVG) for someone who suddenly gets sick?
9. What do you think about the healthcare that is available in SVG for someone who needs to see a doctor for a rash?
10. What would make you more likely to use the district clinics for your current complaint in the future?
11. Is there anything that you ‘like’ about the AED department that makes you more comfortable with the AED?

Interview guides in qualitative research aim to cover pertinent topics but allow for flexible answering of the questions and give the participant the opportunity to place greater emphasis on particular issues over others. A pilot of three interviews was conducted to ascertain if the main researcher (SK-B), a former local emergency physician perhaps known to some participants, might have a noticeable effect on participants’ responses. However, this seemed not the case with answers and interaction open and frank; the pilot interviews were included in the main analysis. Verbatim transcription of the interviews commenced immediately and continued even as new data were collected. The qualitative research software ATLAS.ti V.7 was used to facilitate the coding and organisation of data, grouping responses (see table 2) and allowing for a comparison of the various responses and identification of patterns.

**Table 2:** Coding table

Categories grouped by theme	Frequency
Theme: habitual use of the AED	
Describes automatic/habitual behaviour	7
Difficulty answering questions (short phrases) regarding roles/functions of AED, unable to differentiate between the roles of AED and district clinics	4
Widely shared practice	4
Socially encouraged	7
Total	22
Theme: health system (private and public) encouraged or initiated use of AED	
Clinic schedule	5
Type of staff/doctor seeking	3
Belief that district clinic staff refers patient to AED	2
Dissatisfaction with the behaviour of clinic staff	1
Free service at AED	2
Total	13
Theme: deliberate use of AED	
Based on convenience	6
Based on patients' assessed seriousness of their complaint	4
Past positive AED experiences	3
Despite negative experiences and reports	3
Confidence in AED	4
No cost	2
Familiarity with AED	1
Seeking a doctor	4
Total	27

AED; accident and emergency department.

We collected and analysed the data using a grounded theory approach. The iterative process comprised interviewing, transcribing, preliminary analysis and team reflection, revisions to some interview questions or probes, further interviewing and so forth. Revisions to the interview guide following this constant comparison and exploration meant reconsidering the rational choice model of decision-making towards exploring approaches of habit formation and socially shaped decision-making. For example, later interviews also asked who might have influenced attitudes about the AED within their social world. Interviews stopped when a reasonable point of saturation was reached and no new insights were forthcoming. The continued discussion of emerging ideas with the senior author (CG) aimed to ensure rigour in the data analysis and credibility of findings. Findings have also been shared with local research and health service provider colleagues to allow further critique.

## Results

Three major themes were identified: habitual use of the AED, systemic encouragement of the use of the AED and deliberate use of the AED. In the following quotations, the term ‘hospital’ refers to the AED and the term ‘clinic’ refers to the primary care district clinic.

### Habitual use of the AED

We found that the choice to seek medical care at the AED rather than at a primary healthcare facility often did not involve a deliberate decision-making process but was a default option for the participants, shared by others and encouraged by others. Talking about their personal practices, participant 6 remarked that whenever they were ill, “so then the first place you go turn is here [the AED]”.

This concept of automatically—without reflection—seeking care at the AED seemed to apply to others in our participants’ social world. AED as a default option was thought to be a typical health-seeking response within society. When asked where they personally sought care, the answer often described more general societal behaviour: Going “down at AED; everybody run to Kingstown [location of AED]” (participant 2). “…Most people don't go [to the primary care] clinic. They come hospital [AED] for everything” (participant 7). Participant 11 explained that the convenience of locality did not matter in this case: “Because everybody leave, no matter what part of Saint Vincent, and you take in, and whatever little thing…everybody coming, is here they heading”.

Family and friends encouraged this practice. Participant 10 took advice from *Tanti* [auntie] when deciding to come to AED:

I heard people saying so. And the last time I got stung by one [a centipede] on my belly. I tell me Tanti [auntie], and she say [said] that it poisonous…. Say [said] you have to go casualty….

The same participant also recalled following the recommendation of a supervisor at work to ‘go casualty’, though the initial reaction was to seek a simple bandage: “I put on a rag and go to the supervisor and tell him and ask him if he got any plaster. He say no, you have to go casualty now”.

### Systemic encouragement to use the AED

This habitual use of the AED was at least partially encouraged and reinforced by the healthcare system in SVG. The limited scheduling of doctor-run clinics and the limited hours of functioning of district clinics was noted by participants and encouraged their self-referral to the AED.

I: “How come you didn't go to the district doctor today”?

P5: (pause) “Well, today is not doctor day in my area, so”.

While this kind of self-referral seemed common, participants also reported that they had been frequently referred to AED by district clinic staff. Participant 2, referring to the nurse at the local clinic Belair, said: “You go to Belair now right, becau’ the doctor ain't ‘tening [attending], immediately the nurses at Belair right, is going to send you straight to AED”.

Participant 2 also explained that it was not only the absence of attending doctors but also the absence of diagnostic facilities that further necessitated seeking care elsewhere, and said “…if they have to refer to hospital whether to do a test or so, then the district [nurse] will send you here to do like a ultrasound because they don't do that in district clinics, right”. Participant 7 summed it up by saying, “Why people come [to] the emergency all hour of the day, all hour of the night? Because the district clinic is not…adequate enough”. This also seemed to be a complaint about the private healthcare system: “If you go to a private doctor, pay your money, yes they give you medication, and most of the time you still end up with a letter to come to AED” (participant 2).

### Deliberate use of the AED

Despite many narratives of habitual use, we found some deliberate consideration, to varying degrees, involved in the decision to come to AED, even in those participants who displayed features of habitual use. This deliberate use went beyond the participants’ reaction to systemic unavailability of services. In some cases, conscious reasoning was pragmatically based on convenience. For participant 4, who works in Kingstown, it was a matter of their physical location at the time of onset of the complaint, and said “Most of the time I get headache and I at work, I will come here because is in town”. Participant 7 explained that the decision was based on transportation: “My transport was coming in town. I didn't thought of another clinic at the time”.

Some participants thought that their particular complaint warranted an AED visit. Participant 7 described the reasoning process: “If it's serious, then it mean’ you come down hospital”.

P6: “Because, err, it was an emergency”.

I: “You thought it was an emergency? How did you decide it was an emergency”?

P6: “Because I'm feeling a lot of pain, (barely audible) for six weeks”.

Participants also reported positive experiences with the AED that affected their choice, including quality of care and time available by staff. Participant 11 said, “I know they have very good doctors here [at AED] so, I ain't say they ain't have good doctors outside, but there are very good doctors here that I feel comfortable”. Participant 6 commented on the time spent and extent of care received at the AED compared with the district clinic and commented on it, “sometimes up there [local clinic] they don't take time with you…”. Participant 5 felt that the perception of better care at the AED was one held by the general public, and said, “but people, if you ask me my opinion, people just want to, I don't know, people probably think you will get a better service at the, err, casualty”.

## Discussion

In this qualitative study on the attitudes that influence the non-urgent use of the AED, we found that locally shared attitudes shaped a habitual use of the AED, reinforced by the structure and operations of the healthcare system in SVG and by some deliberate choice to visit the AED such as convenience. This study echoed widely reported factors of convenience, cost, access to facilities and dissatisfaction with the primary care providers.^10^ ^11^ ^16^ Much of the existing body of research on health services usage and particularly the AED has focused on these convenience and access issues referred to as logistical aspects in the Anderson framework of assessing health services use.^21^ The research design of this study, therefore, has been initially guided by social cognitive theories and rational theories of decision-making, intention and behaviour, assuming that non-urgent AED use is based on the rational assessment of the patient of the seriousness of their complaint, along with issues of convenience and availability of services.^14^

Although the healthcare system in SVG differs from those in high-income settings, Vincentians face similar concerns, particularly as their US counterparts; they try to access healthcare that is highly rationalised and where physician care and diagnostic facilities are not easily available.^10^ AED was not understood to be uniquely designed for life-threatening complaints. Rather, some participants saw AED as a place where the doctor or nurse would always be and hence the appropriate place for providing care, not because of the specialised services available there.

While some participants contemplated the nature of the complaint, the overt perception of need based on the seriousness of their complaint was not as prominent in this study on Vincentian non-urgent patients compared with the North American or European patients.^9^ ^22^ Where participants in this study understood AED to be solely for emergencies, the perceptions of what constituted an emergency were quite different to those of healthcare providers. Therefore, participants were confident that theirs was the right decision to seek emergency-level care. This discrepancy is an observation reported by other researchers.^8^

An unexpected finding of our study was that participants overwhelmingly narrated AED use as a socially shared habit of ‘going casualty’. To our knowledge, this is the first study that found and explored AED use as shaped by habit or custom rather than solely based on deliberate decision-making or outweighing of barriers, and socially imitated or shared within their families and communities. That said, this habitual behaviour was found to be at least partially created by the system that offers limited access to doctors. Although none of the participants in this study had been referred on the visit when the interview occurred, most had experience of being referred by healthcare providers to the AED because of lacking physician, facility or equipment availability. Social learning and enhancement also seemed to have occurred when family, friends and authority figures such as employers passed on the recommendation of ‘going casualty’.

The study's findings make clear that to understand non-urgent AED use in this setting we need to acknowledge this interplay of habitual use, social and systemic encouragement, and rational outweighing of best healthcare access, as proposed in Zimmerman's^23^ multilevel theory of population health. In research on population and patient behaviour, social cognitive theories, though successful at determining intentions as indicators of behaviour in longitudinal studies, failed to explain 50–60% of the variance in actual behaviour relative to intention.^24^ Nilsen *et al*^24^ propose that habit might be better placed to explain behaviours and could improve behaviour change interventions that to date fail to show meaningful success. Moving one step further, Zimmerman^23^ suggests to go beyond intrapersonal understandings of habit and to understand that habits are often socially widely imitated and turn into ‘customs’, and are intrinsically embedded in power structures such as the healthcare systems in which patients find themselves. This study presents one of the first empirical examples of applying Zimmerman's multilevel framework of health behaviours.

### Study limitations

As the main limitation of this research, we note that this was a small study conducted within the constraints of a postgraduate degree programme and set in a small setting. To make this study feasible, we had to limit the participant group to a relatively homogenous sample to reach reasonable saturation within a relatively small sample size. We therefore excluded parents seeking help for their children and also healthcare professionals. Attitudes and practices of healthcare professionals explored in this study were solely narrated by participating patients. Perhaps the most important limitation of this study was to only include non-urgent attendees of the AED, although the analysis suggested that interviewing the population outside the AED setting could have added valuable insight on ‘going casualty’ as a socially shared custom. We would also recommend conducting a follow-up quantitative study using a measure of habit such as the Self-Report Habit Index to inform any health service interventions.^25^ Finally, as any qualitative study, these findings are highly context-specific and we recognise that this study has been carried out in a small setting. Nonetheless, we hope that our study speaks to many similar resource-poor settings, and more generally, that it can contribute towards a growing body of research that aims to understand socially shared and structured health and health-seeking behaviours in a variety of settings and topics.

Combining understandings of health behaviour as habits, and more importantly socially shared customs, shaped by and negotiated within healthcare structures, suggests a better explanation of the observed behaviour than the more commonly used rational theory model in health services research. This novel way of understanding the decision-making process, in this case of the non-urgent AED patient, potentially allows for the design of interventions that can more effectively address the upstream components that determine the use of the AED.
